# Delayed-Onset Hypoglycemia After Levofloxacin Use in a Diabetic Patient

**DOI:** 10.1155/carm/3220993

**Published:** 2025-12-01

**Authors:** Henry Dwaah, Sarah Tupchong, Joseph Kim

**Affiliations:** Montefiore Medical Center, 111 E 210th St, Bronx 10467, New York, USA

## Abstract

We discuss the case of a patient with diabetes on long-term glipizide and stable glucose management who presented with delayed-onset hypoglycemia 6 weeks after initiating levofloxacin for osteomyelitis. The patient presented with neuroglycopenic symptoms, including dysarthria, slurred speech, and syncopal episodes. Serum glucose level was 35 mg/dL, and C-peptide was markedly elevated at 12.42 ng/mL. Hypoglycemia resolved immediately after D50 administration. Discontinuation of glipizide while on levofloxacin did not result in any new hypoglycemic episodes. In previously documented cases, hypoglycemia occurred within days or less than a week after initiating levofloxacin. This report represents an interesting case of delayed-onset hypoglycemia after initiating levofloxacin in a stably managed diabetic patient on long-term glipizide secretagogue.

## 1. Introduction

Hypoglycemia is a serious adverse event that complicates the management of diabetes mellitus [1]. This complication commonly occurs with concurrent fluoroquinolone and insulin or sulfonylurea therapy and can lead to worsening morbidity and mortality, especially in older populations [2, 3]. Medication-induced hypoglycemia requires a high index of suspicion in patients previously on stable long-term glucose control and started on fluoroquinolone) [3, 4]. Cases of hypoglycemia associated with fluoroquinolones have been well-documented [5, 6]. While these cases of hypoglycemia occurred acutely within a short time span of drug introduction, we present a case of delayed hypoglycemia after long-term levofloxacin use in a patient on glipizide sulfonylurea.

## 2. Case Presentation

A 70-year-old man with medical history significant for hypertension, CKDII, osteomyelitis of the fifth metatarsal of the left foot, and Type 2 diabetes mellitus presented to our institution complaining of 1-week history of intermittent slurred speech and syncopal episodes. He arrived dysarthric and confused. He reported feeling dizzy with leg weakness and inability to speak right before passing out. He had a total of three syncopal episodes within a week before presenting to the hospital. He denied any head strike but noted several bruises on his body. In the previous syncopal episodes, he had received orange and apple juice which appeared to help. He also reported that in the past month, he had felt more unsteady on his feet along with generalized weakness. The patient denied any substance and alcohol use. He denied any headache, shortness of breath, chest pain, or abdominal pain. Patient reported some chronic mild pain in the left foot. Further history revealed that 2 months prior to presentation, he had been admitted to the hospital for osteomyelitis of the fifth metatarsal of the left foot. At that time, amputation of the fifth metatarsal was recommended, but he declined and elected for conservative management with antibiotic therapy. He was started on levofloxacin 750 mg oral daily for 8 weeks. He was approximately 6 weeks into his antibiotic course when he presented to the emergency department (ED) with syncopal episodes.

### 2.1. Investigations

In the ED, he was afebrile, with the heart rate of 82, respiratory rate of 18, blood pressure of 123/59, and SpO_2_ of 96% on room air. Laboratory workup revealed a serum glucose of 35 mg/dL and C-peptide of 12.42 ng/mL. Insulin levels were not sent at that time. Creatinine was stably elevated at 1.6 (baseline 1.5), GFR 59 (range 55–63 in prior months), potassium 3.8, and sodium 137. Complete blood count (CBC) had normal white count and hemoglobin of 12, AST < 20, ALT of 10, albumin of 3.8, and total bilirubin of 0.5. Urine toxicology was negative. Ethanol, salicylate, and acetaminophen levels were normal. CT head was negative for acute infarct. Troponins were negative 2 times, and EKG had sinus rhythm with known first-degree A-V block; however, new premature supraventricular complexes were present. X-ray of the left foot showed osteomyelitis of the fifth metatarsal, overall unchanged from previous admission.

### 2.2. Differential Diagnosis

Presentation of dysarthria, confusion, and syncope raised concerns for stroke, fluid and electrolyte derangement, infections, drug (salicylates and barbiturates) or alcohol toxicity, low cardiac perfusion states (heart failure and heart block), and metabolic derangements (hypoglycemia, adrenal insufficiency, and so on). Acute infarct, electrolyte derangements, and cardiac etiologies were quickly ruled out. Infection and worsening osteomyelitis were considered; however, patient was afebrile, not tachycardia, and no leukocytosis on labs. Additionally, blood cultures remained negative, and his x-ray foot did not suggest any progression of osteomyelitis on current antibiotics. The patient's low glucose, however, pointed towards metabolic derangement. His history of Type II diabetes suggested insulin or insulin secretagogue involvement. The patient had been on long standing glipizide therapy, and thus C-peptide levels were assessed which was markedly elevated ∼ 12.5 ng/mL (normal 0.5–2 ng/mL), suggesting involvement of secretagogue in his hypoglycemic episode.

### 2.3. Treatment

In the ED, the patient received D50 solution with immediate resolution of his presenting symptoms. His home glipizide was discontinued, and he was placed on insulin basal and bolus regimen. Levofloxacin was continued for empiric treatment of his left fifth metatarsal osteomyelitis.

### 2.4. Outcome and Follow-up

On the medical floor, the patient was monitored for recurrence of hypoglycemia to guide further work up. No hypoglycemic episodes were recorded during his 2-week stay after glipizide discontinuation. He also underwent amputation of the left fifth metatarsal during that time. Medication interaction between levofloxacin and glipizide was suspected to be the cause of hypoglycemia. Postoperative, the patient was put on 12-day regimen of levofloxacin and doxycycline for possible residual osteomyelitis. Levofloxacin was continued due to prior history of *Pseudomonas aeruginosa*. Glipizide remained held, and he was discharged on metformin with 2-week follow-up with his primary care physician and infectious diseases. The patient was instructed to discuss with primary care physician on transitioning from metformin to glipizide after completion his antibiotic course.

## 3. Discussion

In this case, the patient presented with symptoms of slurred speech, dysarthria, and syncope, which were found to be due to severe hypoglycemia. The patient's neuroglycopenic symptoms resolved immediately after administering glucose, suggesting a pathologic cause of hypoglycemia as described by Whipple's triad. Given his medical history of Type II diabetes, an elevated endogenous insulin level was believed to be the cause of hypoglycemia, and this was demonstrated by the significantly elevated C-peptide levels, 12.5 ng/mL (normal 0.5–2 ng/mL). The patient has been on long-term glipizide and metformin for glucose control; thus, it was crucial to discontinue these medications in order to assess for other causes of hyperinsulinism, such as insulinoma. The patient underwent a fast to demonstrate Whipple's triad; however, blood glucose levels consistently remained > 70 in the first 24 h. Given that the patient was on concurrent glipizide and levofloxacin therapy, the etiology of the hyperinsulinism was suggested to be medication-related. Additional risk factors that made this diagnosis more likely were his advanced age and mild-to-moderately decreased renal function, which predispose him to hypoglycemic complications while on both sulfonylurea and fluoroquinolone. Of note, sepsis was high on differential diagnosis; however, the patient remained afebrile, normotensive, and without leukocytosis or positive cultures on labs.

Although hypoglycemia is a known adverse effect of sulfonylureas, the risk of hypoglycemia is more commonly observed in drug–drug interactions than when used alone [4, 6, 7]. This patient had been on glipizide therapy for several years with good glycemic control, A1c 6, without any documented episode of hypoglycemia. He, however, began to experience hypoglycemia 6 weeks after beginning a course of levofloxacin while on his long-term glipizide medication. The fluoroquinolone class of medications, which includes levofloxacin, has been shown to induce severe hypoglycemia when used in combination with antihyperglycemics, leading to hospitalization and even death [8, 9]. Among the quinolone class, levofloxacin has been identified as one with increased risk of severe hypoglycemic episode [10].

Fluoroquinolones are highly effective antibacterials for coverage of a wide spectrum of penicillin-sensitive or resistant organisms and anaerobes. Their potent antibacterial qualities make them useful for diabetes-related infections caused by organisms such as *Pseudomonas aeruginosa*. Adverse events commonly associated with the fluoroquinolones include central nervous system toxicity, ECG abnormalities (QT interval prolongation), tendon and joint disorders, and disruption of glucose metabolism [11]. Alternative classes of antibiotics used in diabetes-related infections including macrolides (clarithromycin or azithromycin), oxazolidinone (Linezolid), tetracyclines (tigecycline or doxycycline), and sulfonamide/folate inhibitor (trimethoprim-sulfamethoxazole) have all been associated with hypoglycemia; however, their individual reported risks are relatively lower compared to fluoroquinolones [4, 7, 12]. Patient risk factors for hypoglycemia include age greater than 65, female sex, and prior multiple comorbidities [6].

Fluoroquinolones stimulate insulin release by blocking pancreatic β-cell ATP-sensitive K channels, which trigger β-cell depolarization and insulin release [2, 7, 11, 12]. Sulfonylureas bind to receptor subunits on the same K channels, which results in calcium release and membrane depolarization leading to insulin release [2, 10]. The synergistic effect of both levofloxacin and glipizide on beta cells thus increases the risk of hypoglycemia, especially in elderly patients. Documented cases of quinolone-associated hypoglycemia in diabetic patients occurred within a few days or a week after the introduction of the quinolone with concurrent glipizide or glyburide use [5, 8, 13]. It is interesting that this case of hypoglycemia presented in a delayed fashion, approximately 6 weeks, compared to cases in the literature. It suggests the interplay of other key mechanisms in the risk of hypoglycemia that are yet to be discovered. Differences in binding interaction between sulfonylurea and its subunit on the K channel (discussed below) may be implicated in this delayed hypoglycemic onset. Further research will be needed to fully understand the factors at play in the onset of acute versus delayed levofloxacin-associated hypoglycemia in diabetics on long-term secretagogue therapy.

Sulfonylurea is primarily bound to albumin, 98% to 99% while levofloxacin is bound 24% to 38%. Both drugs bind at different sites on albumin and have minimal influence on each other's binding affinities. Their limited interaction while bound to albumin is not adequate to explain the increased risk of hypoglycemia in patients. Genetic variants of the sulfonylurea subunits of ATP-sensitive K channels may likely shed light on the delayed onset of hypoglycemia [14]. The effectiveness of drug–protein binding interactions is altered due to polymorphism in the gene encoding the ATP-sensitive K channels, and this changes insulin response to sulfonylurea treatment [14]. Lastly, sulfonylureas are metabolized via hepatic CYP2C9 enzymes, which have different functional variants as a result of mutations in alleles. These functional variants, if present, may also alter the availability of active metabolites of sulfonylurea and impact the risk of hypoglycemia [15].

There have been reports of rebound hypoglycemia in patients after treatment of hypoglycemia with dextrose or glucagon despite discontinuation of sulfonylurea [2]. One proposed mechanism is that dextrose serves as a potent stimulus for additional insulin release in sulfonylurea-exposed patients with intact pancreatic function (e.g., noninsulin-dependent Type 2 diabetes) [2]. Although dextrose is given to treat hypoglycemia, it also triggers beta cells to release insulin, which can complicate hypoglycemic episodes, making recovery challenging. A second proposed mechanism is the delayed half-life of sulfonylurea, especially in patients with chronic kidney disease, which can cause prolonged hypoglycemic episodes despite treatment with dextrose. Sulfonylurea half-life is usually 2–5 h in patients with optimal renal function. The presence of renal disease may prolong the excretion of sulfonylurea active metabolites, which can increase the risk of hypoglycemia. Our patient did not experience any rebound hypoglycemic episodes, likely due to relatively optimal renal function (GFR 59) and early resumption of a carbohydrate-consistent clear liquid diet after mental status recovery following initial D50 bolus. The patient's glucose was monitored closely after glipizide discontinuation, and he was counseled to increase oral sugar consumption during hospitalization to prevent further hypoglycemic episodes.

In summary, we present the case of a patient with optimal glucose control on long-term glipizide therapy who has new delayed-onset hypoglycemic episodes 6 weeks after initiating levofloxacin for osteomyelitis management. This case highlights the importance of having a high index of suspicion for drug–drug interaction–associated hypoglycemia in previously well-managed diabetic patients started on quinolones, especially levofloxacin, and with new hypoglycemic episodes. This case shows that hypoglycemic episodes may not only occur within the first few days but may also occur weeks or months after initiating quinolone therapy with concurrent use of glipizide therapy. Further studies are warranted to explore the incidence of late-onset hypoglycemia in diabetic patients on concurrent fluoroquinolone and sulfonylurea therapy.

## Data Availability

The data supporting the findings of this case report are fully described within the manuscript and are available from the corresponding author upon reasonable request, with appropriate consideration for patient privacy.
